# Differences in Lifestyle Improvements With the Intention to Prevent Cardiovascular Diseases by Socioeconomic Status in a Representative Japanese Population: NIPPON DATA2010

**DOI:** 10.2188/jea.JE20170254

**Published:** 2018-03-05

**Authors:** Sayuri Goryoda, Nobuo Nishi, Atsushi Hozawa, Katsushi Yoshita, Yusuke Arai, Keiko Kondo, Naoko Miyagawa, Takehito Hayakawa, Akira Fujiyoshi, Aya Kadota, Takayoshi Ohkubo, Tomonori Okamura, Nagako Okuda, Hirotsugu Ueshima, Akira Okayama, Katsuyuki Miura

**Affiliations:** 1 The Disease Prevention Science Course, Graduate School of Medical and Dental Science, Tokyo Medical and Dental University, Tokyo, Japan; 2 International Center for Nutrition and Information, National Institute of Health and Nutrition, National Institutes of Biomedical Innovation, Health and Nutrition, Tokyo, Japan; 3 Department of Preventive Medicine and Epidemiology, Tohoku Medical Megabank Organization, Tohoku University, Miyagi, Japan; 4 Department of Food and Human Health Science, Osaka City University Graduate School of Human Life Science, Osaka, Japan; 5 Department of Nutrition, Chiba Prefectural University of Health Sciences, Chiba, Japan; 6 Department of Public Health, Shiga University of Medical Science, Shiga, Japan; 7 Research Center for Social Studies of Health and Community, Ritsumeikan University, Kyoto, Japan; 8 Center for Epidemiologic Research in Asia, Shiga University of Medical Science, Shiga, Japan; 9 Department of Hygiene and Public Health, Teikyo University School of Medicine, Tokyo, Japan; 10 Department of Preventive Medicine and Public Health, Keio University School of Medicine, Shinjuku, Tokyo, Japan; 11 Department of Health and Nutrition, University of Human Arts and Sciences, Saitama, Japan; 12 Research Institute of Strategy for Prevention, Tokyo, Japan

**Keywords:** healthy behaviors, socioeconomic status, lifestyle, eating habits, dietary habits

## Abstract

**Background:**

The relationships among socioeconomic status and lifestyle improvements have not yet been examined in a representative Japanese population.

**Methods:**

We analyzed data from 2,647 participants (1,087 men and 1,560 women) who participated in NIPPON DATA2010. This survey inquired about lifestyle improvements and socioeconomic status. Education was categorized as low (≤9 years), middle (10–12 years), and high (≥13 years). Marital status was categorized as married, divorced, widowed, and never married/other. A multivariable logistic regression model was used to calculate the odds ratios (ORs) and 95% confidence intervals (CIs) of lifestyle improvements with the intention of preventing cardiovascular diseases for educational attainment and marital status, with adjustments for age and awareness of cardiovascular disease risk factors.

**Results:**

Overall, 1,507 (56.9%) participants practiced prevention and improvements in hypertension, diabetes, elevated cholesterol, and metabolic syndrome, and the OR of lifestyle improvements was significantly higher with a high education than with a low education in men (OR 2.86; 95% CI, 1.96–4.17) and women (OR 2.36; 95% CI, 1.67–3.33). The number of participants who practiced prevention and improvements in hypertension, diabetes, elevated cholesterol, and metabolic syndrome was significantly lower in divorced than in married men (OR 0.46; 95% CI, 0.22–0.95) and women (OR 0.53; 95% CI, 0.33–0.86).

**Conclusions:**

Specific differences caused by educational attainment and marital status may exist in lifestyle improvements.

## INTRODUCTION

Differences in aspects of socioeconomic status (SES), such as marital status and educational attainment, are associated with the incidence of cardiovascular diseases (CVD).^1^^–^^3^ SES and risk factors for CVD have mainly been reported in Europe and the United States, with few studies being conducted in Japan. Findings on the relationships among SES and healthy behaviors have been inconsistent.^4^^,^^5^ In a study conducted on adult participants with low SES in the Netherlands, the perception of healthy eating was triggered by the existence of a partner or children living together.^4^ In another study in Ireland, no relationships were found among healthy behaviors, education level, and marital status.^5^ In Japan, unhealthy lifestyles, such as unhealthy eating habits and insufficient exercise, were reported to be related to lower SES^6^; however, the relationships among SES and lifestyle improvements with the intention of preventing CVDs, including metabolic syndrome, have not yet been investigated. In this study, we used a baseline survey dataset of a representative Japanese population to examine relationships among socioeconomic factors, including educational attainment and marital status, in consideration of the awareness of CVD risk factors with lifestyle improvements for the intention of preventing CVDs. The effects of SES on relationships among lifestyle improvements and attitudes regarding several healthy behaviors were also investigated.

## METHODS

### Study population

The National Integrated Project for Prospective Observation of Non-communicable Disease and its Trends in the Aged 2010 (NIPPON DATA2010) was established in 2010 as a prospective cohort study to investigate factors associated with the morbidity and mortality of CVDs in Japan. Details are provided elsewhere.^7^^–^^9^ In summary, the baseline survey was conducted at the same time as the National Health and Nutrition Survey of Japan in 2010 (NHNS2010)^10^ in November 2010. Participants of NHNS2010 were residents aged 1 year and older living in 300 survey districts throughout Japan. Participants aged 20 years and older and who were given blood tests were invited to enroll in NIPPON DATA2010. Trained interviewers obtained informed consent individually. Participants in NHNS2010 had also participated in the Comprehensive Survey of Living Conditions 2010 (CSLC2010), which was conducted in June 2010 (before NHNS2010). Data obtained from the NIPPON DATA2010 baseline survey were merged with those obtained from NHNS2010 and CSLC2010.

### Lifestyle improvements

Lifestyle improvements were examined using the lifestyle questionnaire for NHNS2010. A participant was considered to be practicing lifestyle improvements if he/she answered yes to “Do you practice lifestyle improvements with the intention of preventing CVDs (prevention and control of hypertension, diabetes, elevated cholesterol, or metabolic syndrome)?”.

### Attention to healthy behaviors

Attention to healthy behaviors was also investigated using the lifestyle questionnaire for NHNS2010. Participants were asked if they paid attention in their daily life to the following seven healthy behaviors: avoid overeating, reduce salt intake, avoid too much fat, avoid too many sweets, eat more vegetables, eat more fish than meat, and do more exercise.

### Educational attainment

Information on educational attainment was obtained from the NIPPON DATA2010 baseline questionnaire. Participants were asked of their highest level of education by selecting one of five levels: none (did not graduate from any school), elementary school (6 years), junior high school (3 years), high school (3 years), junior college including professional school (2–3 years), and university including graduate school (4 years and more). Educational attainment was grouped into three categories using total educational years: low (≤9 years), middle (10–12 years), and high (≥13 years). In Japan, elementary school and junior high school are compulsory.

### Marital status

Marital status was assessed from the NIPPON DATA2010 baseline questionnaire. Participants were asked, “What is your current marital status?”. Answer options were: married, divorced, widowed, never married, and other.

### Awareness of CVD risk factors

Information on awareness of CVD risk factors was obtained from the lifestyle questionnaire for NHNS2010. A participant was considered to be aware of hypertension if he/she answered yes to the question “Have you ever been told that you were hypertensive at a medical facility or a medical checkup?”. Regarding awareness of diabetes mellitus, participants were asked “Have you ever been told that you had diabetes mellitus (or ‘borderline diabetes’, were ‘likely to become diabetic’, or had ‘high blood sugar levels’)?”. Concerning awareness of hypercholesterolemia, participants were asked if they have been told that they had “high blood cholesterol levels (total cholesterol or LDL cholesterol)”.

### Statistical analysis

A total of 2,891 participants were enrolled in NIPPON DATA2010. Our analytic sample comprised 2,647 individuals (1,087 men and 1,560 women), after excluding those with a previous history of stroke (*n* = 109), myocardial infarction (*n* = 51), or angina (*n* = 90), or those with missing variables (*n* = 29). All statistical analyses were performed on men and women separately. We created dummy variables for age, SES, and awareness of CVD risk factors and used them in a multivariate analysis; age groups (20–29, 30–39, 40–49, 50–59, 60–69, 70–79, and ≥80 years [reference]), education (high, middle, and low [reference]), marital status (married [reference], divorced, widowed, and never married/other), awareness of hypertension (yes and no [reference]), diabetes mellitus (yes and no [reference]), and hypercholesterolemia (yes and no [reference]).

In order to assess relationships among lifestyle improvements and SES, a multivariate logistic regression analysis was used, with age groups, education, marital status, and awareness of hypertension, diabetes mellitus, and hypercholesterolemia as independent variables and lifestyle improvements as the dependent variable. Attention to healthy behaviors was compared for those with lifestyle improvements and others using chi-squared tests. In order to assess relationships between attention to healthy behaviors and SES, a multivariate logistic regression analysis was used, with age groups, education, marital status, and awareness of hypertension, diabetes mellitus, and hypercholesterolemia as independent variables and lifestyle improvements as the dependent variable. Analyses were performed for each of the seven healthy behaviors. All analyses were conducted using SPSS version 24 (IBM Corp, Armonk, NY, USA), and *P* values less than 0.05 were considered to be significant.

### Ethical considerations

This study was approved by the Institutional Ethics Committee of Shiga University of Medical Science.

## RESULTS

Table 1 summarizes the basic characteristics and lifestyle improvements of the study participants. Of the total sample, 24.7% of men and 23.3% of women answered that their education level was low (≤9 years), and 3.3% of men and 5.4% of women were divorced. Regarding CVD risk factors, 37.1% of men and 29.6% of women were aware of hypertension, 16.2% of men and 8.8% of women were aware of diabetes, and 34.4% of men and 36.3% of women were aware of hypercholesterolemia. Younger participants and those who were divorced, never married, or had a low education level were less likely to practice lifestyle improvements for the prevention and control of CVD risk factors. On the other hand, among men and women, married participants and those who were aware of any CVD risk factors practiced lifestyle improvements more than others.

**Table 1.** Characteristics of participants: NIPPON DATA2010 (*n* = 2,647)

	Men (*n* = 1,087)		Women (*n* = 1,560)	
	
	Total (%)	Lifestyle improvements (%)^a^	Total (%)	Lifestyle improvements (%)^a^
Number		53.0		59.7
Age, years				
Mean (SD)	58.8 (15.8)	60.6 (14.7)	57.2 (16.0)	59.1 (14.8)
Age group, years				
20–29	5.2	33.3	4.8	36.0
30–39	9.9	43.5	15.0	49.6
40–49	11.3	47.2	11.4	51.7
50–59	16.2	52.8	17.6	62.2
60–69	29.7	58.2	26.3	67.4
70–79	20.2	59.1	19.0	64.5
≥80	7.4	51.3	5.8	62.6
Education, years				
High (≥13)	33.6	59.2	31.3	58.9
Middle (10–12)	41.8	53.3	45.4	63.4
Low (≤9)	24.7	44.0	23.3	53.4
Marital status				
Married	81.0	54.4	73.4	61.3
Divorced	3.3	44.4	5.4	44.7
Widowed	4.0	54.4	12.9	64.4
Never married/Other	11.6	45.2	8.2	47.7
Awareness of CVD risk factors, answered “Yes”				
Hypertension	37.1	65.5	29.6	74.7
Diabetes	16.2	73.9	8.8	81.0
Hypercholesterolemia	34.4	66.0	36.3	72.8

CVD, cardiovascular disease; SD, standard deviation.

^a^Defined as participants who answered, “yes” to the question “Do you practice lifestyle improvements with the intention of preventing CVDs (prevention and control of hypertension, diabetes, elevated cholesterol, or metabolic syndrome)?”.

Odds ratios (ORs) and 95% confidence intervals (CIs) for lifestyle improvements obtained from multivariate logistic regression analyses are shown in Table 2. Older participants were more likely to practice lifestyle improvements for the prevention and control of CVD risk factors. Education levels were significantly associated with lifestyle improvements in men and women, independent of marital status and awareness of CVD risk factors. Significantly more men with high and middle education levels practiced lifestyle improvements than those with a low education level; the OR was 2.86 (95% CI, 1.96–4.17) for a high education level and 1.86 (95% CI, 1.32–2.63) for a middle education level. The same relationships were observed among women; the OR was 2.36 (95% CI, 1.67–3.33) for a high education level and 2.15 (95% CI, 1.60–2.88) for a middle education level.

**Table 2.** Odds ratios and 95% confidence intervals of answering “yes” to the question of lifestyle improvements in the multivariable logistic regression analysis: NIPPON DATA2010

	Men (*n* = 1,087)		Women (*n* = 1,560)	
	
	OR	95% CI	OR	95% CI
Age group, years				
20–29	0.45	0.19–1.06	0.54	0.25–1.18
30–39	0.65	0.33–1.27	0.78	0.43–1.42
40–49	0.65	0.34–1.25	0.80	0.43–1.47
50–59	0.73	0.40–1.34	1.03	0.58–1.83
60–69	1.09	0.63–1.88	1.17	0.69–1.98
70–79	1.23	0.70–2.14	1.03	0.61–1.74
≥80	1.00	(reference)	1.00	(reference)
Education, years				
High (≥13)	2.86	1.96–4.17	2.36	1.67–3.33
Middle (10–12)	1.86	1.32–2.63	2.15	1.60–2.88
Low (≤9)	1.00	(reference)	1.00	(reference)
Marital status				
Married	1.00	(reference)	1.00	(reference)
Divorced	0.46	0.22–0.95	0.53	0.33–0.86
Widowed	0.82	0.43–1.59	0.94	0.65–1.36
Never married/Other	1.16	0.73–1.82	0.88	0.56–1.38
Awareness of CVD risk factors, answered “Yes”				
Hypertension	1.98	1.49–2.63	2.15	1.62–2.84
Diabetes	2.46	1.67–3.62	2.17	1.36–3.46
Hypercholesterolemia	1.83	1.38–2.43	1.99	1.55–2.55

CI, confidence interval; CVD, cardiovascular disease; OR, odds ratio.

Regarding marital status, divorced men and women followed lifestyle improvements significantly less than those living with their spouses, independent of other SES and awareness of CVD risk factors. No significant associations were observed between the bereavement of a spouse or remaining unmarried and lifestyle improvements over living with spouses. Significantly more participants who were aware of CVD risk factors practiced lifestyle improvements than others among both men and women.

Table 3 shows the percentages of participants who paid attention to healthy behaviors according to lifestyle improvements. Participants practicing lifestyle improvements were significantly more attentive to all healthy behaviors than those not practicing lifestyle improvements in both men and women.

**Table 3.** Attention to heathy behaviors among participants who were practicing lifestyle improvements with the intention of preventing CVDs “Yes” or “No”: NIPPON DATA2010

	Men					Women				
	Lifestyle improvements					Lifestyle improvements				
	
	Yes (*n* = 576)		No (*n* = 511)			Yes (*n* = 931)		No (*n* = 629)		
	*n*	%	*n*	%	*P*-value	*n*	%	*n*	%	*P*-value
Attentive of not eating too much.^a^	368	63.9	151	29.5	<0.001	588	63.2	208	33.1	<0.001
Attentive of reducing salt.^a^	304	52.8	131	25.6	<0.001	602	64.7	217	34.5	<0.001
Attentive of not eating too much fat.^a^	317	55.0	116	22.7	<0.001	623	66.9	217	34.5	<0.001
Attentive of not eating too many sweets.^a^	258	55.0	100	19.6	<0.001	515	55.3	146	23.2	<0.001
Attentive of eating more vegetables.^a^	357	62.0	165	32.3	<0.001	662	71.1	291	46.3	<0.001
Attentive of eating more fish than meat.^a^	268	46.5	124	24.3	<0.001	532	57.1	176	28.0	<0.001
Attentive of doing more exercise.^a^	349	60.6	123	24.1	<0.001	489	52.5	151	24.0	<0.001

CVDs, cardiovascular diseases.

^a^Defined as participants who answered “yes” to each question on healthy behaviors.

Among the seven healthy behaviors, “eating more fish rather than meat” was less prevalent in men than the other behaviors. In women, “eating more vegetables” was more prevalent among those practicing lifestyle improvements (71.1%), while approximately half (46.3%) of those not practicing lifestyle improvements were attentive to it.

Table 4 shows the results of the multivariate logistic regression analysis using attention to each healthy behavior as an objective variable and socioeconomic factors as explanatory variables, with adjustments for age and awareness of CVD risk factors. Younger participants were more likely to be less attentive to all healthy behaviors than older participants (data not shown). Participants with a high or middle education level were significantly more attentive to doing more exercise than those with a low education level. Regarding other behaviors, no significant associations were observed with education level. Among men, unmarried men were more attentive “to reducing salt intake” and “not eating too much fat”, while widowed men were less attentive to “not eating too many sweets”. Widowed women were less attentive to “not eating too much fat”. Awareness of CVD risk factors was associated with the corresponding health behaviors.

**Table 4.** Odds ratios and 95% confidence intervals of answering “yes” to each question on healthy behaviors in the multivariable logistic regression analysis: NIPPON DATA2010

	Men (*n* = 1,087)		Women (*n* = 1,560)	
	
	OR	95% CI	OR	95% CI
“Attentive of not eating too much.”				
Education, years				
High (≥13)	0.99	0.69–1.42	1.34	0.97–1.85
Middle (10–12)	1.02	0.74–1.43	1.26	0.96–1.66
Low (≤9)	1.00	(reference)	1.00	(reference)
Marital status				
Married	1.00	(reference)	1.00	(reference)
Divorced	0.79	0.40–1.57	0.94	0.60–1.49
Widowed	0.92	0.49–1.73	0.75	0.53–1.06
Never married/Other	1.16	0.74–1.82	0.82	0.52–1.28

“Attentive of reducing salt.”				
Education, years				
High (≥13)	0.97	0.67–1.40	1.12	0.80–1.56
Middle (10–12)	1.02	0.72–1.43	1.16	0.87–1.54
Low (≤9)	1.00	(reference)	1.00	(reference)
Marital status				
Married	1.00	(reference)	1.00	(reference)
Divorced	0.59	0.28–1.23	0.66	0.41–1.06
Widowed	0.71	0.37–1.34	0.82	0.57–1.17
Never married/Other	2.40	1.45–3.98	1.14	0.72–1.80

“Attentive of not eating too much fat”				
Education, years				
High (≥13)	1.42	0.98–2.05	1.38	0.99–1.92
Middle (10–12)	1.38	0.99–1.93	1.22	0.92–1.62
Low (≤9)	1.00	(reference)	1.00	(reference)
Marital status				
Married	1.00	(reference)	1.00	(reference)
Divorced	1.27	0.64–2.53	0.67	0.42–1.07
Widowed	0.74	0.39–1.39	0.68	0.48–0.96
Never married/Other	2.55	1.56–4.17	0.68	0.43–1.08

“Attentive of not eating too many sweets.”				
Education, years				
High (≥13)	1.27	0.87–1.85	1.01	0.73–1.41
Middle (10–12)	1.06	0.74–1.50	0.97	0.74–1.28
Low (≤9)	1.00	(reference)	1.00	(reference)
Marital status				
Married	1.00	(reference)	1.00	(reference)
Divorced	0.62	0.29–1.32	0.70	0.43–1.14
Widowed	0.47	0.23–0.97	0.78	0.55–1.10
Never married/Other	1.87	1.15–3.04	0.89	0.56–1.43

“Attentive of eating more vegetables.”				
Education, years				
High (≥13)	1.34	0.94–1.92	1.20	0.86–1.67
Middle (10–12)	1.03	0.74–1.43	1.22	0.92–1.62
Low (≤9)	1.00	(reference)	1.00	(reference)
Marital status				
Married	1.00	(reference)	1.00	(reference)
Divorced	0.60	0.30–1.21	0.98	0.62–1.56
Widowed	0.87	0.46–1.62	0.74	0.51–1.05
Never married/Other	0.95	0.60–1.50	0.80	0.52–1.24

“Attentive of eating more fish than meat.”				
Education, years				
High (≥13)	1.36	0.94–1.96	1.14	0.82–1.59
Middle (10–12)	1.19	0.85–1.67	1.09	0.83–1.44
Low (≤9)	1.00	(reference)	1.00	(reference)
Marital status				
Married	1.00	(reference)	1.00	(reference)
Divorced	1.42	0.72–2.81	0.85	0.53–1.36
Widowed	0.89	0.47–1.66	0.75	0.54–1.06
Never married/Other	1.61	0.98–2.62	0.70	0.43–1.12

“Attentive of doing more exercise.”				
Education, years				
High (≥13)	2.03	1.41–2.93	1.48	1.06–2.06
Middle (10–12)	1.61	1.15–2.25	1.43	1.09–1.89
Low (≤9)	1.00	(reference)	1.00	(reference)
Marital status				
Married	1.00	(reference)	1.00	(reference)
Divorced	0.94	0.47–1.89	1.05	0.66–1.68
Widowed	0.90	0.48–1.70	0.83	0.59–1.16
Never married/Other	1.27	0.81–2.00	1.15	0.72–1.84

CI, confidence interval; OR, odds ratio.

ORs are adjusted for age, awareness of hypertension, diabetes, and hypercholesterolemia.

Socioeconomic factors interact with each other. We performed a multivariate logistic regression analysis using <100,000 JPY/month or ≥100,000 JPY/month of equivalent household expenditure (EHE) and found no significant associations with lifestyle improvements or any healthy behavior, with/without other socioeconomic factors (data not shown).

## DISCUSSION

This study revealed some differences in lifestyle improvements for the prevention and control of CVD risk factors and related aspects of SES: higher engagement in lifestyle improvements in men and women with high educational backgrounds and lower engagement in divorced men and women, after adjustments with covariates including age and awareness of CVD risk factors. To the best of our knowledge, this is the first study to investigate relationships among SES and lifestyle improvements with the intention of preventing CVD in a representative Japanese population.

Regarding the relationship between overall lifestyle improvements and education level, similar findings have been reported in a previous study.^6^ A low education level and lower household income were also reported to be associated with a higher salt intake in a cross-sectional study on workers in a Japanese manufacturing plant.^11^ We found no significant associations between education levels and attention to reducing salt in the present analysis. A preference for salty food may be different among individuals with different educational backgrounds, and this may also be attributable to job category. In this study, lifestyle improvements were less common among divorced men and women than married men and women. In previous longitudinal studies in the United States, the detrimental effects of the loss of a marital relationship on diet, particularly in terms of decreased vegetable intake, were more prominent among divorced/widowed men^12^ and women^13^ than those who remained married. Decreases in average BMI were also observed for divorced/widowed participants in these studies.^12^^,^^13^ In a cross-sectional study in Japan, unhealthy behaviors, such as poor dietary habits, were more strongly associated with divorced men than those who remained married.^14^ In the present study, no significant associations were observed between marital status and attention to “not eating too much” in men and women, whereas widowed men were less attentive to “not eating too many sweets” and widowed women were less attentive to “not eating too much fat”, which may have been caused by a decreased appetite. Previous studies in Japan indicated that widowed or divorced women have a higher risk of CVD.^15^^,^^16^ Our results on reluctance regarding lifestyle improvements in divorced men and women and indications of the loss of appetite among widows/widowers may be partially attributable to unfavorable CVD risk factors in these groups.

The present study focused on differences in lifestyle improvements and attention to healthy behaviors among those in various social sectors, and the results obtained indicate that appropriate care needs to be provided to individuals with low and middle education levels and to divorced/widowed adults, who may not be as conscious as married adults of lifestyle improvements and may develop poor dietary habits.

Our study has several limitations. A number of socioeconomic factors may be associated with health, and these factors may be related to one another. We only investigated a limited number of socioeconomic factors in this study. The relationships found here may be different when adjusted for other aspects of SES that may confound the associations. Furthermore, economic factors may change in a relatively short period. Further careful investigations are needed in order to elucidate the relationships among economic factors, including income and working situation, and lifestyle improvements.

### Conclusion

We conclude that certain differences caused by education and marital status exist in lifestyle improvements with the intention to prevent CVDs, even after adjusting for factors such as age and awareness of CVD risk factors.
